# DNA methylation mediates BmDeaf1-regulated tissue- and stage-specific expression of *BmCHSA*-*2b* in the silkworm, *Bombyx mori*

**DOI:** 10.1186/s13072-018-0202-4

**Published:** 2018-06-14

**Authors:** Guanfeng Xu, Jie Zhang, Hao Lyu, Qisheng Song, Qili Feng, Hui Xiang, Sichun Zheng

**Affiliations:** 1 Guangzhou Key Laboratory of Insect Development Regulation and Applied Research, Institute of Insect Science and Technology, School of Life Sciences, South China Normal University, Guangzhou, 510631 China; 2 Division of Plant Sciences, College of Agriculture, Food and Natural Resources, University of Missouri, Columbia, MO 65211 USA

**Keywords:** DNA methylation, Intragenic promoter, Transcriptional regulation, Chitin synthase, Wing development

## Abstract

**Background:**

Accurate regulation of tissue- and stage-specific expression of genes is prerequisite for normal development in organisms. DNA methylation plays an important role in modulating gene expression in mammals and plants. However, there is no direct evidence showing how DNA methylation regulates gene transcription in insects.

**Results:**

During the development of *Bombyx mori* wing, the expression level of DNA methyltransferase 1 (BmDnmt1) gradually declined and became stationary at pupal stage, resulting in a lower methylation rate of the intragenic promoter of the mid-pupal wing-specific gene *BmCHSA*-*2b*, an epidermal chitin synthase controlling mid-pupal wing development in *B. mori*. The higher methylation rate of the promoter in the pupal epidermis was decreased and *BmCHSA*-*2b* transcription was significantly increased by the treatment with the DNA methylation inhibitor, 5-azacytidine-2′-deoxycytidine, suggesting that DNA methylation regulates the tissue-specific expression of *BmCHSA*-*2b*. Pupa-specific transcription factor BmDEAF1 bound to the unmethylated intragenic promoter and activated the *BmCHSA*-*2b* transcription in the mid-pupal wing. BmDnmt1 and BmDeaf1 influenced the *BmCHSA*-*2b* transcription by binding competitively to the CpG island in the promoter.

**Conclusions:**

All the data together demonstrate that the cooperation between the down-regulation of BmDnmt1 and increased stage-specific expression of BmDeaf1 enhances *BmCHSA*-*2b* tissue- and stage-specific transcription to ensure mid-wing development in *B. mori*. This study highlights an elaborate regulation mechanism how tissue- and stage-specific gene expression is regulated through promoter methylation in insect development.

**Electronic supplementary material:**

The online version of this article (10.1186/s13072-018-0202-4) contains supplementary material, which is available to authorized users.

## Background

DNA methylation is a covalent modification that targets the fifth carbon of the pyrimidine ring of cytosines, which is catalyzed by DNA methyltransferase to give rise to 5-methylcytosine (5-mC) in genomic DNA [1, 2]. DNA methylation has been studied extensively in mammals and plants [3]. In mammals, about 60–90% of CpGs are methylated across entire genomes [4] and DNA methylation-mediated transcriptional regulation usually occurs at promoter regions and telomeres [5, 6] to create the binding sites for specific transcription factors to activate the expression of some tissue-specific genes [7] or inhibit the transcription factor binding to the promoter, resulting in altered transcriptional activity of the gene. DNA methylation is involved in genomic imprinting, X-chromosome inactivation, silencing of transposons and other repetitive DNA sequences, in particular, the inhibitory regulation of gene expression [8].

In invertebrates, the study of DNA methylation is rudimentary, compared to that in mammals and plants [9]. In insects, DNA methylation in the model insect fruit fly is rather elusive [10, 11] and overall extremely low levels of DNA methylation were observed in silkworm and honeybee [12, 13]. Bioinformatics analyses revealed that different methylation rates were associated to specific phenotypes, such as wing differentiation in *Sogatella furcifera* [14], caste differentiation [15] and long-term memory formation in *Apis mellifera* [16]. It is speculated that the DNA methylation occurs mainly in gene body regions and enhances gene transcription while promoter methylation is often considered not to be involved in the regulation of gene transcription because of its lower methylation rate in insects [17]. Recently, it is observed that DNA methylation in the gene promoter of the invertebrate, *Ciona intestinalis*, was tissue- and/or cell-type specific [18], similar to those identified in mammals. In *Drosophila* S2 cells, up-regulated promoter methylation rate inhibited the promoter activity of steroidogenic enzyme [19], suggesting the regulatory functionality of DNA methylation in the promoter of insect. However, the direct experimental evidence for the regulatory mechanism of DNA methylation has not been reported in insects.

The wing disks of the silkworm, *B. mori,* an important economic and model insect of *Lepidoptera*, undergo dramatic morphological changes and structural evagination to form pupal wings during larva–pupa transition [20]. Recently, a *B. mori* chitin synthase (*BmCHSA*) was characterized, which catalyzes the synthesis of chitin, a major component of wings and epidermis consisting of polymers of *N*-acetylglucosamine [21, 22]. Two alternative splicing variants of *BmCHSA*, *BmCHSA*-*2a* and *BmCHSA*-*2b* were up-regulated in the beginning and middle of pupal wing, driven by two different promoters, respectively. *BmCHSA*-*2b* RNAi resulted in the undeveloped wing [23]. The intragenic promoter that activates the tissue- and stage-specific expression of *BmCHSA*-*2b* is located between exon 2a and exon 2b of *BmCHSA* [23]. We hypothesized that intragenic promoter methylation mediates the tissue-specific expression of *BmCHSA*-*2b*.

In this study, we revealed that demethylation or unmethylation of CpG island 2 (CpGI2) of the intragenic promoter, as a consequence of DNA methyltransferase 1 (DNMT1) down-regulation, enhanced the binding of pupa-specific transcription factor BmDeaf1 to the unmethylated CpGI2 and activated *BmCHSA*-*2b* transcription in mid-pupal wing, thus demonstrating that intragenic promoter methylation plays an important role in mediating tissue and stage-specific expression of genes in insects.

## Result

### The CpGI2 of *BmCHSA*-*2b* promoter is differentially methylated between the pupal wings and epidermis

**Fig. 1:**
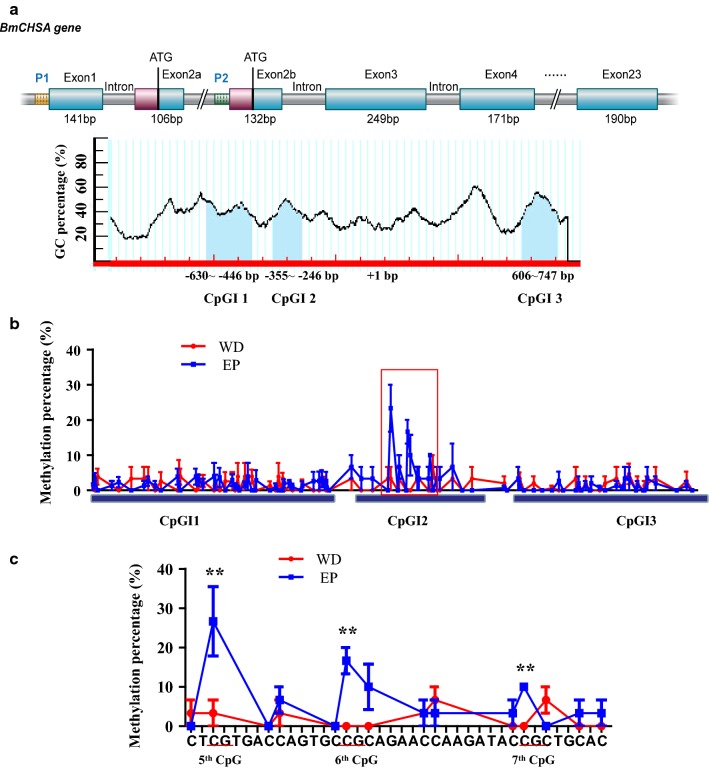
Identification of the methylation sequences in the promoter region of the *BmCHSA*-*2b* gene. **a** *BmCHSA* gene structure showing the positions of two promoters (P1 and P2) and the three CpG islands in the 2 kb of promoter P2 and 5′ untranslated region of *BmCHSA*-*2b*. The blue boxes show the exons and the red boxes show the 5′-UTR of *BmCHSA*-*2a* and *BmCHSA*-*2b.* The tubular lines show the region of the introns and the promoters of *BmCHSA*-*2a* and *BmCHSA*-*2b.* The light blue shapes show the regions of CpGI1, 2 and 3. **b** The methylation rate analysis of CpGI1, 2 and 3 in the *BmCHSA*-*2b* promoter in the 3-day-old pupal wings (WD, red line) and epidermis (EP, blue line) by bisulfite sequencing PCR (BSP). The bolded blue lines show the methylation positions of the CpGI1, 2 and 3. **c** The enlarged and detailed figure of the squared region in **b**, showing the sequence of the detailed nucleotides and the methylation positions in the CpGI2. All data included three biological replicates, each with nine individual repeats. For the *t* test: *p *< 0.05 (*) or *p *< 0.01(**)

*BmCHSA* has two promoters: promoter 1 (P1) and promoter 2 (P2) (Fig. 1a). P1 and P2 control the transcription of *BmCHSA*-*2a* and *BmCHSA*-*2b*, respectively. P2 is located in the intron between exons 2a and 2b. Except the first exon, shares almost the same amino acid sequence with *BmCHSA*-*2a*. Three CpGIs were predicted in the 2 kb of promoter P2 and 5′untranslated region of *BmCHSA*-*2b* using the CpG Island Prediction program [24]: CpGI1 and 2 are located at − 630 ~ − 446 bp and − 355 ~ − 246 bp of the promoter, respectively, while CpGI3 is located at 5′ UTR (606–747 bp) of *BmCHSA*-*2b* transcript (Fig. 1a). The lengths of the three CpGIs are 184, 115 and 141 bp, respectively, and the numbers of CpG are 9, 9 and 12, respectively. *BmCHSA*-*2b* was specifically expressed in the pupal wings [21]. To investigate whether or not the difference in the DNA methylation rates regulates the tissue-specific expression of *BmCHSA*-*2b* in the pupal wings, the methylation rates of CpGI1, 2 and 3 of *BmCHSA*-*2b* promoter in the pupal wings and epidermis were analyzed. The cytosines of unmethylated gDNA isolated from the wings and epidermis of 3-day-old pupae, at which *BmCHSA*-*2b* was up-regulated, were transformed to uracil by bisulfite modification. CpGI1, 2 and 3 were amplified from bisulfite-treated gDNA by PCR and then sequenced by pyrosequencing. The sequencing analysis revealed that hypermethylation occurred at the 5th, 6th and 7th CpG sites in CpGI2 and the methylation rates were significantly higher in pupal epidermis than in pupal wings (Fig. 1b), with 5th CpG site having the highest methylation rate (Fig. 1c). No significant methylation difference was detected in the CpGI1 and 3 between the pupal epidermis and wings (Fig. 1b). This result implied that the highly methylated CpGI2 of P2 in pupal epidermis might be responsible for the suppression the *BmCHSA*-*2b* expression in the pupal epidermis. This CpGI2 fragment (38-mer oligonucleotide duplex including the 5th, 6th and 7th CG sites) of the intragenic promoter P2 was focused for the subsequent investigation.

### BmDnmt1 suppresses the *BmCHSA*-*2b* transcription

**Fig. 2:**
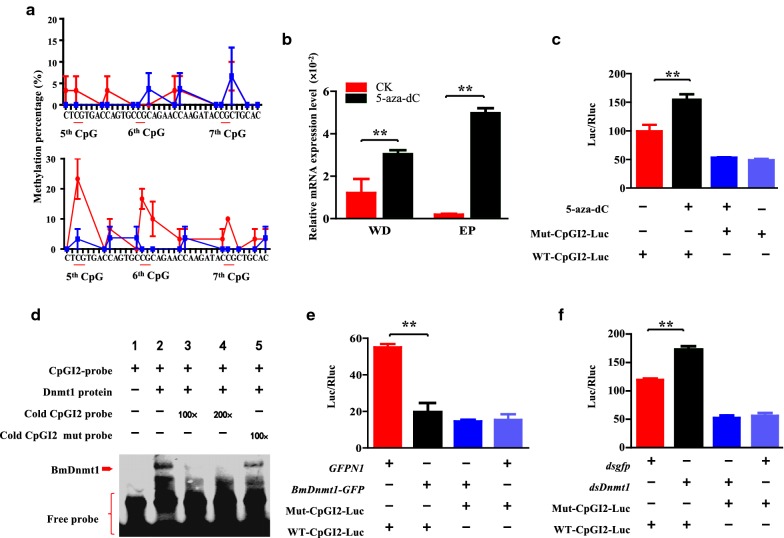
Effects of DNA methylation on the transcription efficiency and promoter activity of *BmCHSA*-*2b*. **a** Effect of methyltransferase inhibitor 5-aza-dC on the methylation level of the CpGI2 in the promoter of *BmCHSA*-*2b* in the 3-day-old pupal wings (top panel) and epidermis (low panel). Red line: PBS treatment, Blue line: 5-aza-dC treatment. **b** Effect of 5-aza-dC treatment on the transcription levels of the *BmCHSA*-*2b* in the 3-day-old pupal wings (WD) and epidermis (EP). **c** Effect of 5-aza-dC treatment on the luciferase activity in the *Bm*12 cells. The cells were transfected with the CpGI2 promoter luciferase vector (pWT-CpGI2-Luc) or the CpGI2-mutated promoter luciferase vector (pMut-CpGI2-Luc) and were added with 1 µL of 1 μg/μL 5-aza-dC. **d** Electrophoretic mobility shift assay (EMSA) showing the binding of BmDnmt1 protein to the CpGI2 probe. The wild-type and mutant probes were labeled with biotin. The cold probe was the unlabeled wild-type CpGI2 probe. **e** Effect of BmDnmt1 overexpression on the luciferase activity in the *Bm*12 cells. The cells were co-transfected with the CpGI2 promoter luciferase vector (pWT-CpGI2-Luc) or the CpGI2-mutated promoter luciferase vector (pMut-CpGI2-Luc) and the BmDnmt1 protein expression vector (pBmDnmt-GFP) or GFP expression vector (pGFPN1, control). (F) Effect of *BmDnmt1* RNAi on the luciferase activity in the *Bm*12 cells. The cells were co-transfected with *dsBmDnmt1* or *dsgfp* (control) and the CpGI2 promoter luciferase vector (pWT-CpGI2-Luc) or the CpGI2-mutated promoter luciferase vector (pMut-CpGI2-Luc), which expresses luciferase under the control of the wild-type or mutant CpGI2 promoter. For the *t* test: *p* < 0.01(**). The sequences of the wild-type and mutant CpGI2 are shown in Additional file 10: Table S1

To investigate whether or not methylation in the CpGI2 of the *BmCHSA*-*2b* intragenic promoter inhibits the gene transcription, the effects of a specific inhibitor (5-aza-dC) of Dnmt on its activity,methylation rate and the transcription level of *BmCHSA*-*2b* were analyzed. After the 5-aza-dC treatment, the activity of BmDnmt was significantly declined (Additional file 1: Fig. S1), resulting in a significant decrease in the methylation rates of the CpGI2 in the wings and epidermis (Fig. 2a) of 3-day-old pupae. Consequently, the mRNA levels of *BmCHSA*-*2b* were significantly increased comparing to the untreated samples, and the *BmCHSA*-*2b* transcript level in the epidermis was higher than that in the wings (Fig. 2b). The alternative splicing variant *BmCHSA*-*2a* transcript level was not affected by 5-aza-dC treatment (Additional file 2: Fig. S2), suggesting that CpGI2 methylation in the intron of *BmCHSA*-*2a* did not regulate its transcription. In the *Bm*12 cells, the transcriptional activity of the CpGI2 was also enhanced by the treatment of 5-aza-dC (Fig. 2c), whereas the transcriptional activity of the mutated CpGI2 (CTAGTGACCAGTGAAGCAGAACAAAGATACAGCTGCAC, where C at the 5th, 6th and 7th CpG sites was changed to A and are underlined) was not affected by the treatment (Fig. 2c). These results indicate that BmDnmt is involved in the methylation in the CpGI2 of the *BmCHSA*-*2b* intragenic promoter, the CpGI2 mutation results in loss of regulation of BmDnmt to the *BmCHSA*-*2b* transcription.

To further confirm whether or not the methylation of CpGI2 is related to BmDnmt1 that directly binds to the CpGI2, electrophoretic mobility shift assay (EMSA) was performed with the biotin-labeled CpGI2 probe and purified BmDnmt1 protein. The results showed that the BmDnmt1 protein bound to the CpGI2 fragment (Fig. 2d, lane 2). The addition of 100 × or 200 × non-labeled probe (cold probe) resulted in the disappearance of the BmDnmt1-bound band (Fig. 2d, lane 3 and 4). However, the mutated unlabeled CpGI2 probe could not compete off the labeled probe (Fig. 2d, lane 5), suggesting that the binding of BmDnmt1 to the CpGI2 was specific. Transfecting *BmDnmt1* into *Bm*12 cells resulted in the overexpression and accumulation of BmDnmt1 in nuclei (Additional file 3: Fig. S3), and the inhibition of the transcriptional activity of the CpGI2 (Fig. 2e). When *BmDnmt1* was knocked down by RNAi (Additional file 4: Fig. S4), the transcriptional activity of the CpGI2 was enhanced (Fig. 2f). The transcriptional activity of the mutated CpGI2 was not affected by either BmDnmt1 overexpression or RNAi. These results suggest that BmDnmt1 suppresses the transcription of *BmCHSA*-*2b* by directly binding to the CpGI2 site.

### BmDeaf1 activates the *BmCHSA*-*2b* transcription

**Fig. 3:**
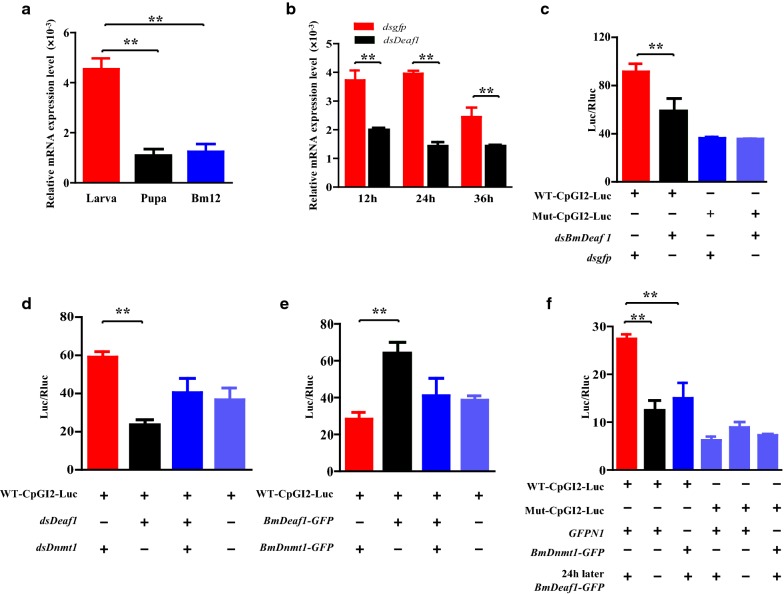
The regulatory effect of BmDeaf1 on *BmCHSA*-*2b* promoter activity. **a** mRNA level of *BmDnmt1* in larvae (Day 3 of fifth instar larval stage), pupae (Day 3 of pupal stage) and the *Bm*12 cell line. **b** Change of *BmDeaf1* mRNA level after *dsBmDeaf1* transfected into the *Bm*12 cells. **c** Changes in the luciferase activities under the control of the wild-type or mutant CpGI2 promoter in the *Bm*12 cells co-transfected with *dsBmDeaf1* or *dsgfp* (as control) and the CpGI2 promoter luciferase vector (pWT-CpGI2-Luc) or the CpGI2-mutated promoter luciferase vector (pMut-CpGI2-Luc). **d** Changes in the luciferase activity under the control of the wild-type CpGI2 promoter in the *Bm*12 cells co-transfected with *dsBmDeaf1*, *dsBmDnmt1* and the CpGI2 promoter luciferase vector (pWT-CpGI2-Luc). **e** Changes in the luciferase activity under the control of the CpGI2 promoter in the *Bm*12 cells co-transfected BmDeaf1-EGFP vector, BmDnmt1-EGFP vector and pWT-CpGI2-Luc. **f** Changes in the luciferase activity under the control of the wild-type or mutated CpGI2 promoter in the *Bm*12 cells co-transfected with pWT-CpGI2-Luc or pMut-CpGI2-Luc and EGFP vector (as control) or BmDnmt1-EGFP vector or BmDnmt1-EGFP vector followed by BmDeaf1-EGFP vector 24 h later. For the *t* test: *p* < 0.01(**). The sequences of the wild-type and mutant CpGI 2 are shown in Additional file 10: Table S1

In order to investigate whether and what transcription factor(s) activates *BmCHSA*-*2b* transcription in the case of low methylation of the CpGI2, *cis*-regulation elements (CRE) in the CpGI2 were analyzed using JASPAR [25, 26]. Five CREs were predicted and Deaf1 CRE in the 5th CpG site has the highest score. *BmDeaf1* was cloned and particularly analyzed. Because in the *Bm*12 cells, *BmDnmt1* expression level was similar to that in the wings of 3-day-old pupae and lower than that in wing disk of 3-day-old fifth instar larvae (5LD3) (Fig. 3a), the cells were used to examine the effect of BmDeaf1 on the CpGI2 transcriptional activity. When the cells were co-transfected with CpGI2-luciferase-expressing vector and *dsBmDeaf1* vector*, BmDeaf1* RNAi significantly inhibited *BmDeaf1* expression (Fig. 3b) and subsequently the transcriptional activity of the CpGI2 (Fig. 3c). When the cells were co-transfected with both *dsBmDnmt1* and *dsBmDeaf1*, the level of the transcriptional activity of the CpGI2 was between *BmDeaf1* RNAi and *dsBmDnmt1* RNAi (Fig. 3d). Overexpression of BmDeaf1 in the *Bm*12 cells resulted in a significant increase in the CpGI2 transcriptional activity, but did not affect the CpGI2 activity when co-transfected with *BmDnmt1* (Fig. 3e) or transfected 24 h post *BmDnmt1* transfection (Fig. 3f). As expected, overexpression of BmDeaf1 had no effect on the mutated CpGI2 activity (Fig. 3f). These results suggest that BmDeaf1 could activate the transcriptional activity of the CpGI2 in the intragenic promoter, when the expression of *BmDnmt1* is inhibited.

### BmDeaf1 directly binds to the unmethylated CpGI2 of *BmCHSA*-*2b*

**Fig. 4:**
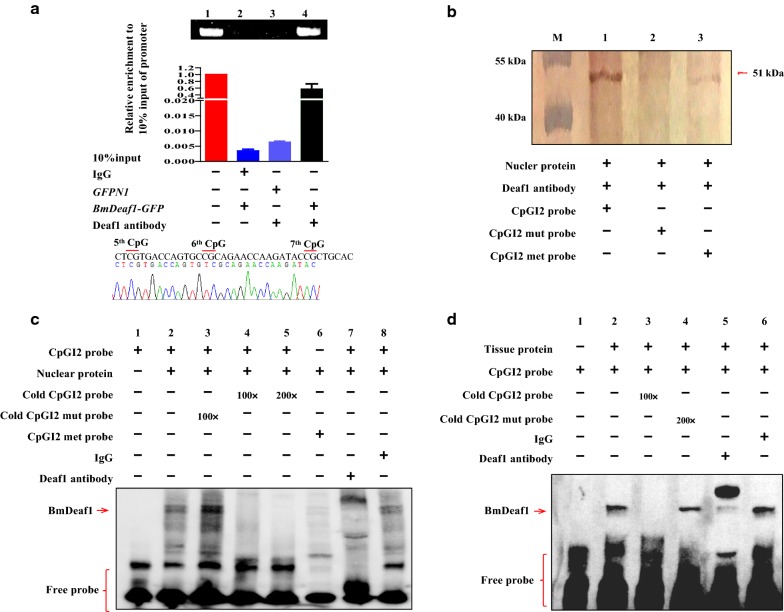
Analysis of the binding of BmDeaf1 with the CpGI2 in the intragenic promoter of *BmCHSA*-*2b*. **a** Analysis of chromatin immunoprecipitation targets detected by RT-PCR (above) and by qRT-PCR (middle) in the *Bm*12 cells. The enrichment of the promoter sequence in the immunoprecipitated DNA samples was normalized with DNA presented in the 10% input material. The enriched RT-PCR product of the ChIP assay was sequenced and aligned with − 270 ~ − 300 in CpGI2 (below). **b** Pull-down experiment with the wild-type or mutant CpGI2 DNA probe and the nuclear proteins from the *Bm*12 cells that were transfected with BmDeaf1-EGFP vector. The proteins that bound to the CpGI2 probe in the supernatant were visualized by Western blot with the BmDeaf1 antibody. The Bio-met-probe: the probe was labeled with biotin and methyl. **c** Electrophoretic mobility shift assay (EMSA) of the binding of the nuclear proteins, which were isolated from the *Bm*12 cells that were transfected with BmDeaf1-EGFP vector, to the CpGI2 probe. The cold probe is the unlabeled CpGI2 probe. The supershifted band was detected by using the BmDeaf1 antibody. **d** EMSA of the binding of the nuclear proteins isolated from the mid-pupal wings to the CpGI2 probe. The supershifted band was detected by using the BmDeaf1 antibody. IgG was used a negative control for the supershift assay. The sequences of the wild-type, mutant and methylated CpGI2 probe are shown in Additional file 10: Table S1

To confirm whether or not the activating effect of BmDeaf1 protein was due to its directly binding to the unmethylated CpGI2 in the *BmCHSA*-*2b* intragenic promoter, chromatin immunoprecipitation (ChIP) experiment was performed with the BmDeaf1 antibody and BmDeaf1 protein expressed in the *Bm*12 cells. The CpGI2 fragment was amplified by PCR with the sample that contained the expressed BmDeaf1 protein and BmDeaf1 antibody (Fig. 4a, lane 4) and the CpGI2 nature of the PCR-amplified sequence was confirmed by sequencing (Fig. 4a). In the samples that contained the BmDeaf1 antibody but no BmDeaf1 protein expressed or the samples that contained BmDeaf1 protein and control (IgG) antibody, there was no CpGI2 PCR product amplified (Fig. 4a, lane 2, 3). To demonstrate whether or not CpGI2 methylation inhibits the binding of BmDeaf1 to the CpGI2, the pull-down assay and EMSA were performed with the methylated or unmethylated CpGI2 fragment (Fig. 4b, c). The result from the pull-down experiment showed that BmDeaf1 bound to the unmethylated CpGI2 fragment, but only a trace amount of binding to the methylated probe was noted (Fig. 4b). A similar result was also observed in the EMSA assay (Fig. 4c). Two strong bands were found on the upper gel with the labeled and unmethylated CpGI2 probe (Fig. 4c, lane 2), which were not present in the control (without the BmDeaf1 protein) (Fig. 4c, lane 1). Addition of 100 × or 200x unlabeled CpGI2 probe (cold probe) resulted in the disappearance of the bands (Fig. 4c, lane 4 and 5). When the probe was mutated, the mutated probe lost the competitive ability with the wild-type CpGI2 probe (Fig. 4c, lane 3). However, the upper strong bands disappeared when labeled and methylated probe was used (Fig. 4c, lane 6). To confirm whether or not BmDeaf1 was in the complex of the upper bands, a supershift assay was performed using the BmDeaf1 antibody. The result showed that the bands were supershifted when the BmDeaf1 antibody was added (Fig. 4c, lane 7), suggesting that BmDeaf1 protein presents in the supershifted bands. No supershifted band was observed when the control IgG antibody was added (Fig. 4c, lane 8).

To confirm whether or not BmDeaf1 protein binds in vivo to the unmethylated CpGI2 in the wings, the nuclear proteins were extracted from the wings of 3-day-old pupae and used for EMSA. The similar result was also obtained (Fig. 4d). A nuclear protein that bound to the labeled unmethylated CpGI2 probe was detected and the binding could be competed off with the unlabeled probe but not with the mutated unlabeled probe (Fig. 4d). A supershifted band appeared after BmDeaf1 antibody was added (Fig. 4d), suggesting that this nuclear protein bound to the unmethylated CpGI2 probe is BmDeaf1. Thus, all of these results from the pull-down assay and EMSA with BmDeaf1 protein demonstrated that in the *Bm*12 cells and the pupal wings, BmDeaf1 bound directly to the unmethylated (but not the methylated) CpGI2.

### The cooperation of BmDnmt1 and BmDeaf1 regulates the stage-specific expression of *BmCHSA*-*2b*

**Fig. 5:**
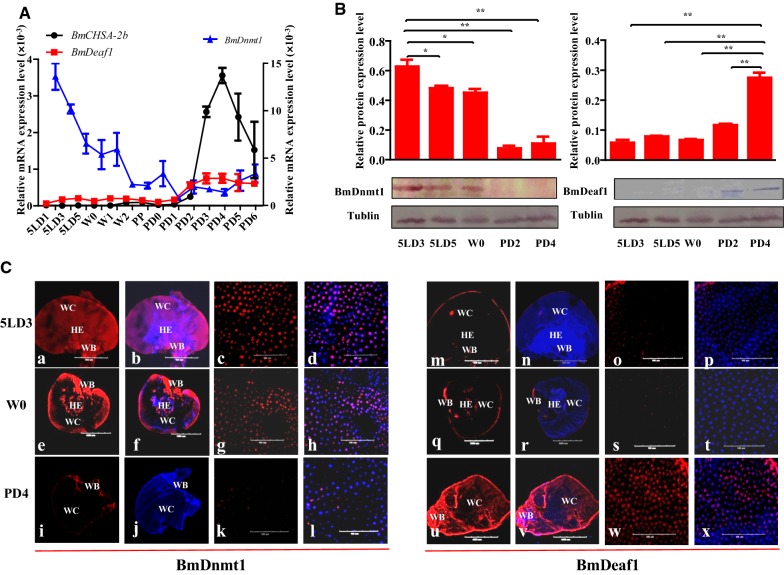
The relationship between the expression of *BmDnmt1*, *BmDeaf1* and *BmCHSA*-*2b* in the wing disk. **A** qRT-PCR analyses of *BmCHSA*-*2b* (black), *BmDnmt1* (blue) and *BmDeaf1* (red) in the wing disk from 1-day-old fifth instar larvae (5LD1) to 6-day-old pupae (PD6). **B** Western blot analyses of BmDnmt1 (left) and BmDeaf1 (right) protein levels in the wing disk at the stages of 5LD3, 5LD5, W0 (the initiation of the wandering stage), PD2 and PD4. The upper panels are the quantitative counts of the bands of target proteins by Western blots in the bottom panels. BmTublin was used to show equal loading of proteins. For the T test: *p *< 0.05 (*) or *p *< 0.01(**). **C** Immunohistochemistry analyses of BmDnmt1 and BmDeaf1 protein levels in the wing disk of fifth instar larvae, wandering larvae and pupa: (a-l) BmDnmt1 protein level at 5LD3 (a-d), W (e–h), PD4 (i-l); (m-x) BmDeaf1 protein level at 5LD3 (m-p), W (q-t), PD4 (u-x). 5LDn: n-day-old fifth instar larvae. *W* wandering stage when the larvae stop feeding, *PDn* n-day-old pupae, *HE* hematopoiesis, *WB* wing base, *WC* wing cell. Scale bar: 500 μm for a–b and m–n; 1 mm for e–f and q–r; 4 mm for i–j and u–v; 100 μm for c–d, g–h, k–l, o–p, s–t and w–x. Blue: DAPI, red in a-l: BmDnmt1 protein, red in m–x: BmDeaf1 protein

To demonstrate whether or not the transcription of *BmCHSA*-*2b* is correlated with the up-regulation of *BmDeaf1* and down-regulation of *BmDnmt1* in vivo, the expression patterns of the three genes in the wing disk from fifth instar to pupal stage were analyzed. *BmDnmt1* mRNA level gradually declined and became stationary from prepupal to the 5-day-old pupal stage, whereas *BmDeaf1* and *BmCHSA*-*2b* were up-regulated starting from 1-day-old pupae and reached a peak at mid-pupa (day 3 and day 4) (Fig. 5A). Western blot analyses showed similar expression patterns of BmDnmt1 and BmDeaf1 to their mRNA expression patterns (Fig. 5B). Immunohistochemistry revealed a similar result as shown in Western blot (Fig. 5Ca–x). When BmDnmt1 level was high before the mid-pupal stage, BmDeaf1 was barely expressed; when BmDnmt1 protein level was significantly decreased in 4-day-old pupal wings, BmDeaf1 was significantly increased. However, BmDnmt1 appeared not to regulate the *BmDeaf1* expression, which was stage-specific but not tissue-specific and it expressed in pupal epidermis (Additional file 5: Fig. S5). The treatment of methyltransferase inhibitor did not affect the expression of *BmDeaf1* but enhanced *BmCHSA*-*2b* transcription at the same stage (Additional files 6, 7: Fig. S6 and S7), suggesting that the stage-specific expression of *BmCHSA*-*2b* is the result of high expression of BmDeaf1, which bound to the unmethylated CpGI2, and low expression of BmDnmt1, which otherwise methylated the CpGI2 and suppressed the BmDeaf1 binding.

## Discussion

*Bombyx mori BmCHSA*-*2b* is a tissue- and stage-specifically expressed gene and expressed only in the pupal wings, not in the epidermis [23]. What controls this high specificity is extremely important for the development progress and, therefore, is interesting. DNA methylation is an epigenetic regulation mechanism for gene expression in specific tissues and at particular stages [27–29]. Many studies have demonstrated that DNA methylation in the promoter region up-stream of the starting site of transcription can regulate the gene transcription [30–33]. Regulation mechanism of gene transcription by DNA methylation in the intragenic promoter for different transcript variants that are tissue- and stage-specifically expressed has been reported in mammals [34], but not in insects. In this study, DNA methylation in the intragenic promoter of *BmCHSA*-*2b* is involved in the regulation of the tissue- and stage-specific expression of the gene in *B. mori* was strongly supported by the critical evidences. The different levels of BmDnmt1 expression in the wing and epidermis at the mid-pupal stage resulted in the different methylation rates in the CpGI2 of *BmCHSA*-*2b* promoter in both tissues and affected the binding of the transcription factor BmDeaf1 to the CpGI2 and finally leaded to the differential expression of gene in both tissues. Thus, our data demonstrated that DNA methylation in the intragenic promoter controls the tissue-specific gene transcription in *B. mori,* in a way similar to that found in mammals [5].

In mammals and plants, the distribution of DNA methylation in genes may appears in two areas: in some cases DNA methylation accumulates in intragenic region; in the others, it appears in the promoter region. DNA methylation in the promoter up-stream of the transcription starting site usually functions as a transcriptional repressor while the methylation in gene body enhances gene transcription [35–37]. In insects, however, the gene body is methylated in much higher rates than the promoter region [17]. The promoter of *BmCHSA*-*2b* is intragenically located in the intron between two parts of the alternative splicing exon 2 of *BmCHSA* (Fig. 1a) and, like the up-stream promoter methylation in mammals, its methylation inhibited the *BmCHSA*-*2b* expression as indicated by results of DNA methylation inhibitor treatment (Fig. 2), *BmDnmt1* RNAi and *BmDnmt1* overexpression (Fig. 2). To investigate whether the DNA methylation in *BmCHSA*-*2b* promoter, which is the intron of *BmCHSA*-*2a*, affects *BmCHSA*-*2a* transcription, *BmCHSA*-*2a* mRNA level was determined after DNA methylation inhibitor treatment and the result showed that *BmCHSA*-*2a* expression was not affected (Additional file 2: Fig. S2), suggesting that the intragenic DNA methylation does not regulate the expression of alternative splicing variant *BmCHSA*-*2a*, instead, as a factor influencing the activity of intragenic promoter and regulating the expression of *BmCHSA*-*2b*. This is similar to the DNA methylation in the intragenic promoters in mammals [34]. In human and mouse *SHANK3* gene, two transcripts (22t and 32t) encode a full-length SHANK3 protein, but they have their own promoters and the shared first exon [34]. The methylation inhibitor treatment only resulted in the increase in 32t transcription. Thus, probably in both insects and mammals, DNA methylation in the intragenic promoter functions as a suppressor to control tissue-specific gene expression. This different effect of intragenic DNA methylation on *BmCHSA*-*2a* and *BmCHSA*-*2b* transcription is coincidence with their expression patterns: *BmCHSA*-*2a* is expressed in pupal wings as well as epidermis while *BmCHSA*-*2b* is specifically expressed in pupal wings [23]. Similar *CHSA* gene structure was also found in other *Lepidoptera*, for example, *Spodoptera litura SlCHSA* [23]*. SlCHSA*-*2b* expression was up-regulated in the mid-pupal wing (Additional file 8: Fig. S8) and was enhanced by methyltransferase inhibitor treatment (Additional file 9: Fig. S9) like *BmCHSA*-*2b* (Additional file 7: Fig. S7). Thus, the DNA methylation occurring in the intragenic promoter probably is one of the mechanisms for regulating the tissue- and stage-specific expression of specific transcripts of an insect gene.

Deaf-1, known as an important transcriptional regulator [38, 39], is ubiquitously expressed and appears to be constitutively localized in nuclei [40]. In this study, BmDeaf1 enhanced *BmCHSA*-*2b* transcription by binding to the unmethylated CpGI2 of *BmCHSA*-*2b* promoter (Fig. 4), as demonstrated using chromatin immunoprecipitation, EMSA and pull-down assays (Fig. 4). Methylated or mutant core sequence TTCG [41] of *Deaf1* CRE in the CpGI3 could not be recognized by BmDeaf1 (Fig. 3). The change of the sequence TTCG into TTAG resulted in the decrease in BmDnmt1 binding (Fig. 2) and increased DNA methylation by BmDnmt1 inhibited the BmDeaf1 enhancement of the *BmCHSA*-*2b* transcriptional activity (Fig. 3), suggesting that BmDnmt1 competes for the same binding site in the CpGI2 with BmDeaf1. However, the methylation inhibitor treatment did not affect *BmDeaf1* expression in the pupal wings and increased the mRNA level of *BmCHSA*-*2b* only in the mid-pupal wings (Additional files 6, 7: Fig. S6 and S7). Furthermore, *BmDeaf1* expression was up-regulated in the pupal epidermis (Additional file 5: Fig. S5) besides in the pupal wing, when the methylation rate in CpGI2 was decreased in the mid-pupal wings but not in the mid-pupal epidermis (Fig. 1), suggesting that the stage-specific expression of BmDeaf1 controls the *BmCHSA*-*2b* expression at the pupal stage by binding to the demethylated CpGI2 site of the *BmCHSA*-*2b* promoter. Deaf-1 was reported to be involved in the development regulation of early embryo, eye and wing in *Drosophila* [40] and could interact with transcriptional regulators LMO4 and NLI to mediate embryonic pattern formation and cell fate, including neuronal differentiation [42, 43]. This transcription factor also regulated immune gene expression, such as Mtk and Drs genes [44]. In *B. mori*, BmCHSA-2b affects the mid-pupal wing development [23]. In this study, it is demonstrated that BmDeaf1 is involved in the regulation of wing development by competing with BmDnmt1 for binding to the CpGI2 of the *BmCHSA*-*2b* promoter.

In animals, DNA methylation is mainly catalyzed by a family of Dnmts, which are classified into Dnmt1, Dnmt2 and Dnmt3 sub-groups. Dnmt3 is primarily responsible for de novo methylation to increase the new methylation sites; the function of Dnmt1 is to maintain the existing methylation [45]. In *B. mori* and *Schistocerca gregaria*, however, Dnmt3 gene is missing and, instead, Dnmt1 enzyme plays a dual function of de novo methylation and the maintenance of methylation in *B. mori* [46, 47]. Binding of BmDnmt1 to 69-mer oligonucleotide duplex containing five CpG sites was also found in *B. mori* [48]. In this study, BmDnmt1 protein directly bound to the 38-mer oligonucleotide duplex containing the 5th, 6th and 7th CpG sites in the CpGI2 promoter of *BmCHSA*-*2b* (Fig. 2d). The 5th CpG region also contains a BmDeaf1 binding site. Thus, overexpression of BmDnmt1 prior to BmDeaf1 inhibited the binding of BmDeaf1 to the CpGI2 and then the BmDeaf1-activated *BmCHSA*-*2b* transcription (Fig. 3). In vivo, BmDnmt1 levels in the wing disk gradually declined from the fifth instar larvae to mid-pupae (Fig. 5). In contrast, the expression of BmDeaf1 was increased so that it bound to the CpGI2 of the *BmCHSA*-*2b* promoter and specifically activated the expression of the gene. This cooperation between BmDnmt1 and BmDeaf1 regulates the tissue- and stage-specific expression of *BmCHSA*-*2b,* as well as the developmental progress. On the other hand, the similar expression profiles of *Dnmt1*, *Deaf1* and *CHSA*-*2b* in *Lepidoptera S. litura* were also observed (Additional file 8: Fig. S8), suggesting that this regulation mechanism of intragenic promoter methylation in *B. mori* pupal wing may be suitable in other insects such as *S. litura*.

## Conclusions

**Fig. 6:**
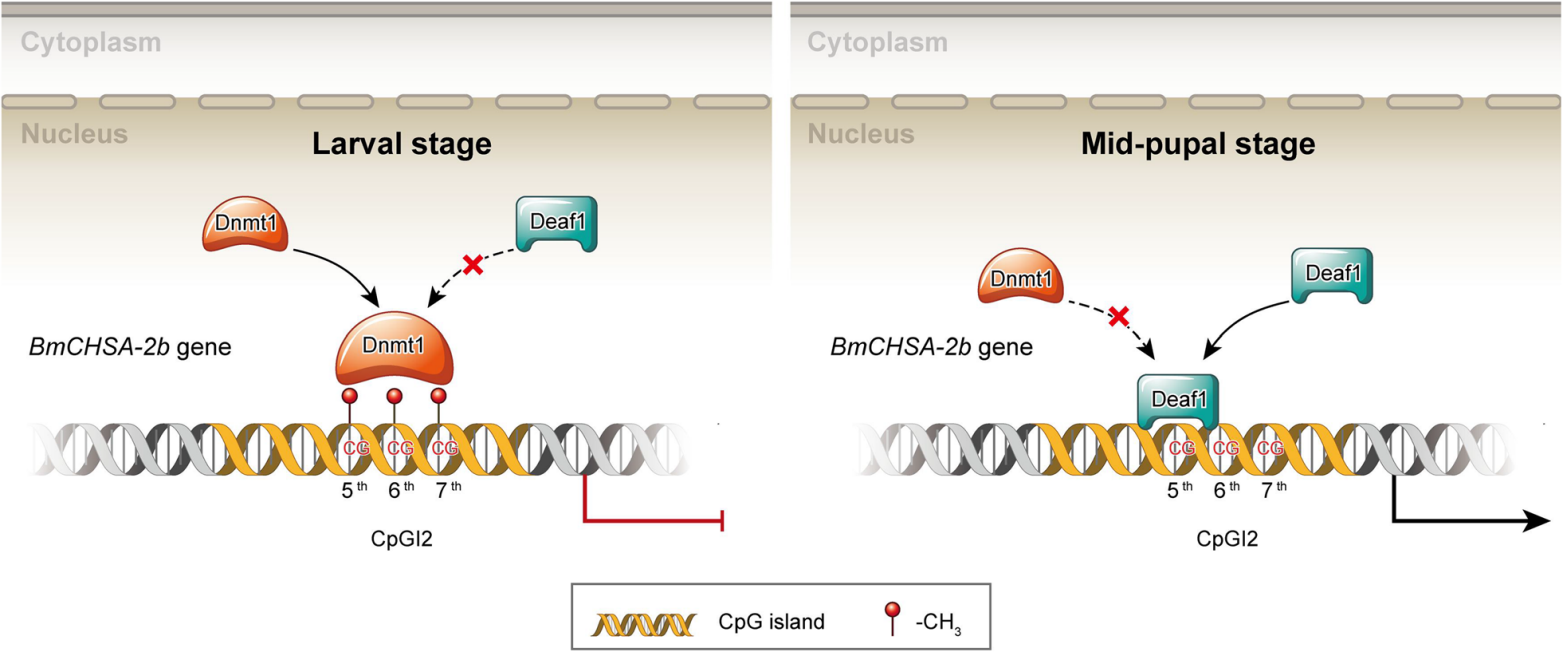
Schematic diagram of the possible regulation mechanism of *BmCHSA*-*2b* tissue- and stage-specific transcription in silkworm. At larval stage, the methyltransferase BmDnmt1 bound to the CpGI2 of the *BmCHSA*-*2b* intragenic promoter and methylated the CpGI2, resulting in the turning-off of the *BmCHSA*-*2b* transcription; during the middle pupal stage, BmDeaf1 bound to the unmethylated CpGI2 and suppressed BmDnmt1 binding to the CpGI2 of the *BmCHSA*-*2b* intragenic promoter, resulting the inhibition of methylation of the CpGI2 and then the turning-on of the *BmCHSA*-*2b* transcription

This study demonstrates that the tissue- and stage-specific expression of *BmCHSA*-*2b* is controlled through the DNA methylation of the CpGI2 of the *BmCHSA*-*2b* intragenic promoter and reveals an elaborate regulation mechanism involving this intragenic promoter methylation in *B. mori* development. Low methylation rate of the intragenic promoter of *BmCHSA*-*2b* in the mid-pupal wing allowed a pupa-specific transcription factor BmDEAF1 to bind with the intragenic promoter and activate the mid-pupal wing-specific gene expression (Fig. 6). These findings provide insights into the regulation of DNA methylation in tissue- and stage-specific gene expression in insects. However, how methyltransferase 1 was down-regulated in the wing during metamorphosis still needs to be explored in the later study.

## Methods

### Insects and cell line

*Bombyx mori* strain Dazao was obtained from the Research and Development Center of the Sericultural Research Institute of the Academy of Agricultural Sciences of Guangdong Province, China. Larvae were reared on fresh mulberry leaves at 25 °C and a photoperiod of 12 h light:12 h darkness.

For methylation inhibitor treatments, 5-aza-dC (Sigma) was dissolved in 1X phosphate buffered saline (PBS). Two microliters of 5-aza-dC at the concentration of 10 μg/μL was injected into the hemolymph in the thoracic region of larvae at the second day of wandering stage (W2), the wings of 2 or 3 day-old pupa (PD2 and PD3) after 5-aza-dC injection were collected for RNA isolation. The same volume of 1× phosphate buffer saline (PBS) was injected as control. All data included three biological replicates, each with three technical repeats.

A *B. mori* cell line DZNU-*Bm*-12 (*Bm*12) originally developed from ovarian tissues [49] was maintained at 28 °C in Grace medium (Invitrogen) supplemented with 10% fetal bovine serum (FBS) (Hyclone).

### Bisulfite conversion and bisulfite sequencing analysis

Genomic DNA of *B. mori* was extracted from pupal wings. Unmethylated cytosines were converted into uracil by using MethylDetector™ (Active Motif, Carlsbad, CA, USA), whereas methylated cytosines remain unchanged. Polymerase chain reaction (PCR) was then performed with primers designed on sequence of CpG islands. Compared to mammals, insects have far lower CpG frequencies. In this study, the region of more than 100 bp with GC% > 50%, Obs/Exp > 0.6 was defined as a CpG island. PCR products were sequenced in order to quantify the level of DNA methylation, which was done by aligning with the sequence of unconverted gDNA using DNAMAN software (Lynnon Biosoft). All data included three biological replicates, each with nine technical repeats.

### RNA isolation and quantitative real-time PCR (qRT-PCR)

Total RNA was extracted from tissue or cell samples using Trizol reagent (TaKaRa), and cDNAs were synthesized by using the First Strand cDNA Synthesis Kit (TaKaRa) following the manufacturer’s protocol. qRT-PCR was performed by using 2 × SYBR Premix EXTaq™ Kit (TaKaRa). The relative mRNA level of gene expression was normalized to the expression level of a house-keeping gene ribosomal protein 49 (Rp49) (GenBank accession no.: AB048205) and analyzed by the 2^−ΔΔCt^ method [50]. All data included three biological replicates.

### Cell culture, transfection and promoter activity determination

*Bm*12 cells at logarithmic growth phase were used for transfection. Plasmid DNAs were mixed with Lipfectamine 2000 (Invitrogen) and added to cells in each well of 12-well culture plates with Grace medium (Invitrogen). To normalize the firefly luciferase activity, the renilla luciferase vector, pRL-SV40, was co-transfected with each of the pGL3-drived vectors containing tested promoters. After 6-h post transfection, the old medium was replaced with fresh Grace medium containing 10% FBS. The cells were cultured for additional 48 h at 28 °C before promoter activity assay. The cells were washed once with filtered PBS and then lysed in 200 μL Passive Lysis Buffer (Promega). Luciferase activity of the supernatant was analyzed using the Dual-Luciferase Assay System (Promega) according to the manufacturer’s instruction with a luminometer (IBA7300, Veritas, Turner Biosystems). All assays were conducted three times.

### Nuclear protein preparation and electrophoretic mobility shift assay (EMSA)

Tissues or cells were harvested and washed three times with PBS. Nuclear protein was extracted according to the instruction of NE-PER Nuclear and Cytoplasmic Extraction Kit (Thermo Scientific, Waltham, USA). EMSA was conducted using the LightShift Chemiluminescent EMSA Kit (Thermo Scientific). The oligonucleotides conjugated with biotin at 5′end or methylated to cytosine were synthesized by Invitrogen. Oligonucleotide probes were heated at 95 °C for 10 min and then slowly cooled to room temperature.

Binding assays were performed according to the manufacture’s protocol of EMSA Kit (Thermo Scientific). Briefly, nuclear extracts (proteins) were incubated for 20 min at room temperature with 20 μL binding buffer containing 50 ng of poly (dI-dC), 2.5% glycerol, 0.05% NP-40, 50 mM potassium chloride, 5 mM magnesium chloride, 4 mM EDTA and 20 fmol of a biotinylated end-labeled double-stranded probe. Different concentrations of cold probes (unlabeled) were added into the binding mixture as competitors. Two micrograms of BmDeaf1 antiserum or 2 μg of normal rabbit IgG (control) was added to detect the supershift bands. Polyacrylamide gels (6%) were run at 100 volts for 1.5 h on ice. After electrophoresis, the proteins were blotted onto positively charged nylon membranes (Hybond Nþ; Amersham Biosciences) and the bands were visualized by using the LightShift Chemiluminescent EMSA Kit according to the manufacturer’s protocol.

### DNA–protein pull-down assays

Single-stranded probes were heated at 95 °C for 10 min and then slowly cooled to room temperature to obtain the double-stranded probes. To minimize non-specific interactions, the oligo-bead complexes were incubated for 30 min with a blocking buffer (0.25% albumin from bovine serum (BSA), 10 mM HEPES, 10 mM glutamate potassium, 2.5 mM DTT, 10 mM magnesium acetate, 5 mM EGTA, 3.5% glycerine, 0.003% NP-40, 0.5% PVP-K30). Immobilized double-stranded probes were incubated with 20 μg of nuclear extract for 4 h at 4 °C with constant rotation in a 400 μL of protein binding buffer (10 mM HEPES, 100 mM glutamate potassium, 80 mM potassium chloride, 2.5 mM DTT, 10 mM magnesium acetate, 5 mM EGTA, 3.5% glycerine, 0.001% NP-40). Protein-DNA complexes were then washed three times with wash buffer (10 mM HEPES, 100 mM glutamate potassium, 2.5 mM DTT, 10 mM magnesium acetate, 5 mM EGTA, 3.5% glycerine, 0.05% BSA, 0.05% NP-40). Proteins bound to the probe were eluted with 20 μL of denaturing Laemmli sample loading buffer (50 mM Tris–HCl, 100 mM DTT, 2% SDS, 0.1% bromophenol blue, 10% glycerine) at 37 °C for 15 min. The target proteins in the supernatant were identified by Western blot with antibody at 1:2000 dilution and the color depth of protein bands was transferred to data by using Image J software (National Institutes of Health, USA).

### Chromatin immunoprecipitation (ChIP)

ChIP was performed in the *Bm*12 cells following the instruction of Pierce Magnetic ChIP Kit (Thermo Scientific). Briefly, approximately 4x10^6^ cells were set up, then cross-linked with 1% formaldehyde for 10 min, and de-cross-linked with glycine. The cells were treated with MNase diluted in MNase Digestion Buffer for 15 min at 37 °C, and then nuclei were released from the cells by using ultrasonic breaking with several pulses and 20 s ice-cold interval. The protein-DNA complexes were immunoprecipitated using antibody, enriched by Magnetic Beads, and cross-linked reversely at 65 °C for 30 min with vigorous rotation. DNA was purified using the column method (Thermo Scientific), and detected by PCR or qPCR. Input (10%) was used as a control.

### DNA methyltransferase activity assay

The nuclear protein at 10 μg extracted from the *Bm*12 cells was treated with 5-aza-dC or PBS (as control). DNA methyltransferase activity was analyzed using the EpiQuik DNA Methyltransferase Activity/Inhibition Assay Kit (Epigentek) following the manufacturer’s instructions. Pure mouse methyltransferase DNMT1 in the kit was used as a positive control. Methyltransferase activity is presented as the average absorbance at 450 nm.

### Western blot analysis

Protein analysis was performed using SDS-PAGE gel and Western blot. Total 40 μg proteins extracted from tissues or *Bm*12 cells were denatured and then separated in 12% SDS-PAGE gel, followed by transferring to a nitrocellulose blotting membrane (GE healthcare). The membrane was blocked with Tris-buffered saline with Tween-20 (TBST) (20 mM Tris–HCl, 150 mM sodium chloride, 0.05% Tween-20, pH7.4) containing 3% (w/v) BSA for 2 h at room temperature, followed by hybridization overnight at 4 °C in TBST containing 1% BSA and primary antibody. The secondary antibody was a horseradish peroxidase (HRP)-conjugated antibody (Dingguo Biotechnology). Primary and secondary antibodies were diluted 1:1000 and 1:10,000 in TBST with 1% (w/v) BSA, respectively. Anti-tubulin antibody (Dingguo Biotechnology) was used to verify equal loading of the proteins on the gel.

### Immunohistochemistry

The newly dissected silkworm tissues or *Bm*12 cells were fixed in 4% paraformaldehyde for 30 or 10 min at room temperature. Tissues or cells were blocked in PBS containing 5% BSA and 0.5% Triton-X (PBT) for 1–2 h, and then incubated with the primary antibody (diluted 1:200) at 4 °C overnight.

Tissues were washed three times for 1 h each in PBT and cells were washed three times for 10 min each. The samples were then incubated with Alexa Fluor™488 goat anti-rabbit IgG (diluted 1:200; Invitrogen) for 2 h. DAPI (Beyotime) was added to stain nucleus. The tissues or cells stained with antibody and DAPI were observed and imaged using a FV3000 confocal microscope (Olympus).

## Additional files

**Additional file 1. Fig. S1.** Effect of the methyltransferase inhibitor 5-aza-dC on the catalytic activity of BmDnmt1 in cell line. *Bm*12 cells were treated with two microliters of 5-aza-dC at the concentration of 1 μg/μL and PBS treatment was used as control.

**Additional file 2. Fig. S2.** Effect of the methyltransferase inhibitor 5-aza-dC treatment on the transcription levels of the *BmCHSA*-*2a.* WD: the 3-day-old pupal wings, EP: the 3-day-old pupal epidermis.

**Additional file 3. Fig. S3.** The nuclear location of BmDnmt1-GFP overexpressed in *B. mori Bm*12 cells (a). GFP (green fluorescent protein) was used as a control. (b). BmDnmt1-GFP. Scale bar: 40 μm. Blue: DAPI.

**Additional file 4. Fig. S4.** RT-PCR (above) and qRT-PCR (below) analyses of *BmDnmt1* mRNA levels post *BmDnmt1* RNAi. The *Bm*12 cells were transfected with *dsBmDnmt1* or *dsgfp* (control). For the *t* test: *p *< 0.05 (*) or *p *< 0.01(**).

**Additional file 5. Fig. S5.** *BmDeaf1* mRNA levels in *B. mori* epidermis from the fifth instar larval to pupal stage. PDn: n-day-old pupae. For the *t* test: *p *< 0.05 (*).

**Additional file 6. Fig. S6.** *BmDeaf1* mRNA levels in the pupal wing treated by the methyltransferase inhibitor 5-aza-dC. Methylation inhibitor 5-aza-dC was injected into the hemolymph in the thoracic region of larvae at the wandering stage, and *BmDeaf1* mRNA levels in the wing at different pupal stages were analyzed. PBS treatment was used as a control. PDn: Day n of pupal stages; P: pupal stage.

**Additional file 7. Fig. S7.** *BmCHSA*-*2b* mRNA levels in the pupal wing treated by the methyltransferase inhibitor 5-aza-dC. Methylation inhibitor 5-aza-dC was injected into hemolymph in the thoracic region of larvae at the wandering stage, and *BmCHSA*-*2b* mRNA levels in the pupal wing were analyzed. PBS treatment was used as a control. PDn: Day n of pupal stages; P: pupal stage. For the *t* test: *p *< 0.01(**).

**Additional file 8. Fig. S8.** mRNA levels of *SlCHSA*-*2b* (black), *SlDnmt1* (blue) and *SlDeaf1* (red) in *S. litura* wing disk. 6LDn: n-day-old sixth instar larvae, PDn: n-day-old pupae, PP: prepupae.

**Additional file 9. Fig. S9.***SlCHSA*-*2b* mRNA levels in the pupal wing treated by the methyltransferase inhibitor 5-aza-dC. Methyltransferase inhibitor 5-aza-dC was injected into hemolymph in the thoracic region of larvae at prepupal stage, and *SlCHSA*-*2b* mRNA levels in the pupal wing were analyzed. PBS treatment was used as a control. PDn: Day n of pupal stages; P: pupal stage. For the *t* test: *p *< 0.05(*).

**Additional file 10. Table. S1.** The sequences of the primers in the study. The underlines represents the methylated sites.

## Abbreviations

BSA: Bovine serum albumin
CHSA: chitin synthase
ChIP: chromatin immunoprecipitation
CpGI: CpG island
CRE: Cis-regulation elements
DAPI: 4′,6-diamidino-2-phenylindole
Deaf1: deformed epidermal autoregulatory factor 1
Dnmt1: DNA methyltransferase 1
DTT: dithiothreitol
EDTA: ethylene diamine tetraacetic acid
EGTA: ethylene glycol bis(2-aminoethyl ether)tetraacetic acid
EMSA: electrophoretic mobility shift assay
FBS: fetal bovine serum
HEPES: *N*-2-hydroxyethylpiperazine-*N*-ethane-sulphonicacid
IgG: immunoglobulin G
MNase: micrococcal nuclease
PBS: phosphate buffer saline
SDS: sodium dodecyl sulfate
TBST: Tris-buffered saline tween-20
UTR: untranslated region
5-mC: 5-methylcytosine
5-aza-dC: 5-azacytidine-2′-deoxycytidine
