# Supplementary figures and images for: DNA methylation mediates BmDeaf1-regulated tissue- and stage-specific expression of BmCHSA-2b in the silkworm, Bombyx mori
Source: Epigenetics Chromatin. 2018 Jun 14;11:32. doi: 10.1186/s13072-018-0202-4 (PMC6001065; doi:10.1186/s13072-018-0202-4)

Fig.S1

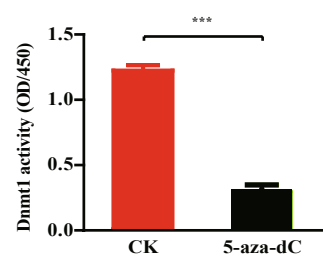

Supplement: Supplementary file 1 — Additional file 1. Fig. S1. Effect of the methyltransferase inhibitor 5-aza-dC on the catalytic activity of BmDnmt1 in cell line. Bm12 cells were treated with two microliters of 5-aza-dC at the concentration of 1 μg/μL and PBS treatment was used as control. [file 13072_2018_202_MOESM1_ESM.pdf]

Fig.S2

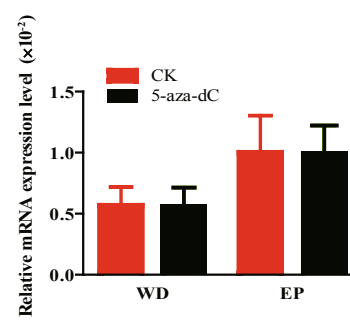

Supplement: Supplementary file 2 — Additional file 2. Fig. S2. Effect of the methyltransferase inhibitor 5-aza-dC treatment on the transcription levels of the BmCHSA-2a. WD: the 3-day-old pupal wings, EP: the 3-day-old pupal epidermis. [file 13072_2018_202_MOESM2_ESM.pdf]

Fig.S3

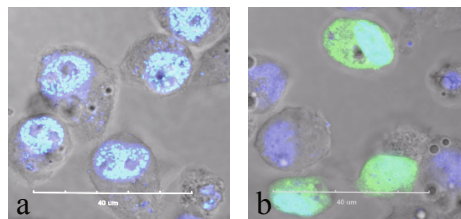

Supplement: Supplementary file 3 — Additional file 3. Fig. S3. The nuclear location of BmDnmt1-GFP overexpressed in B. mori Bm12 cells (a). GFP (green fluorescent protein) was used as a control. (b). BmDnmt1-GFP. Scale bar: 40 μm. Blue: DAPI. [file 13072_2018_202_MOESM3_ESM.pdf]

Fig.S4

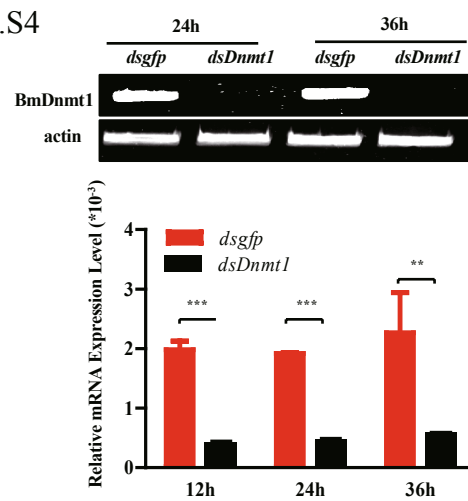

Supplement: Supplementary file 4 — Additional file 4. Fig. S4. RT-PCR (above) and qRT-PCR (below) analyses of BmDnmt1 mRNA levels post BmDnmt1 RNAi. The Bm12 cells were transfected with dsBmDnmt1 or dsgfp (control). For the t test: p < 0.05 (*) or p < 0.01(**). [file 13072_2018_202_MOESM4_ESM.pdf]

Fig.S5

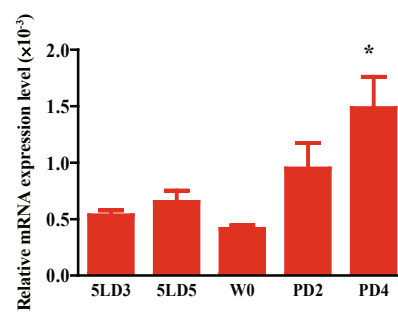

Supplement: Supplementary file 5 — Additional file 5. Fig. S5. BmDeaf1 mRNA levels in B. mori epidermis from the fifth instar larval to pupal stage. PDn: n-day-old pupae. For the t test: p < 0.05 (*). [file 13072_2018_202_MOESM5_ESM.pdf]

Fig.S6

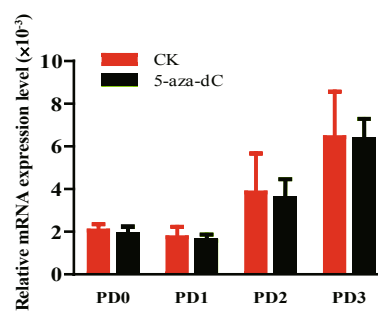

Supplement: Supplementary file 6 — Additional file 6. Fig. S6. BmDeaf1 mRNA levels in the pupal wing treated by the methyltransferase inhibitor 5-aza-dC. Methylation inhibitor 5-aza-dC was injected into the hemolymph in the thoracic region of larvae at the wandering stage, and BmDeaf1 mRNA levels in the wing at different pupal stages were analyzed. PBS treatment was used as a control. PDn: Day n of pupal stages; P: pupal stage. [file 13072_2018_202_MOESM6_ESM.pdf]

Fig.S7

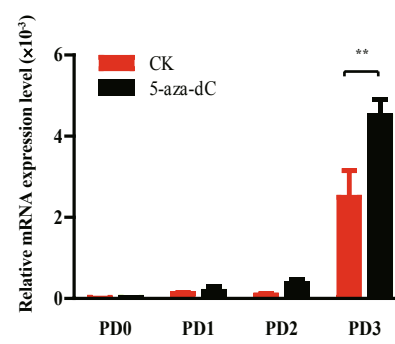

Supplement: Supplementary file 7 — Additional file 7. Fig. S7. BmCHSA-2b mRNA levels in the pupal wing treated by the methyltransferase inhibitor 5-aza-dC. Methylation inhibitor 5-aza-dC was injected into hemolymph in the thoracic region of larvae at the wandering stage, and BmCHSA-2b mRNA levels in the pupal wing were analyzed. PBS treatment was used as a control. PDn: Day n of pupal stages; P: pupal stage. For the t test: p < 0.01(**). [file 13072_2018_202_MOESM7_ESM.pdf]

Fig.S8

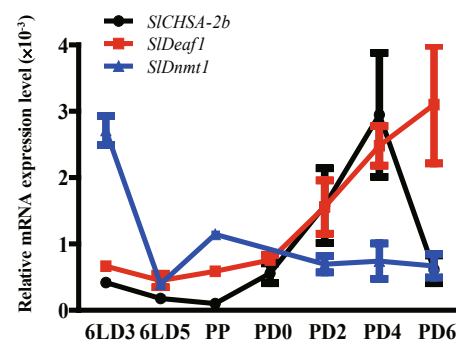

Supplement: Supplementary file 8 — Additional file 8. Fig. S8. mRNA levels of SlCHSA-2b (black), SlDnmt1 (blue) and SlDeaf1 (red) in S. litura wing disk. 6LDn: n-day-old sixth instar larvae, PDn: n-day-old pupae, PP: prepupae. [file 13072_2018_202_MOESM8_ESM.pdf]

Fig.S9

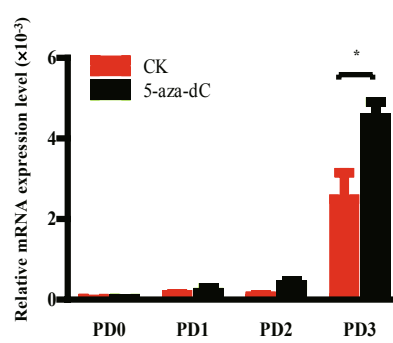

Supplement: Supplementary file 9 — Additional file 9. Fig. S9.SlCHSA-2b mRNA levels in the pupal wing treated by the methyltransferase inhibitor 5-aza-dC. Methyltransferase inhibitor 5-aza-dC was injected into hemolymph in the thoracic region of larvae at prepupal stage, and SlCHSA-2b mRNA levels in the pupal wing were analyzed. PBS treatment was used as a control. PDn: Day n of pupal stages; P: pupal stage. For the t test: p < 0.05(*). [file 13072_2018_202_MOESM9_ESM.pdf]
