# Supplementary material for: DNA methylation mediates BmDeaf1-regulated tissue- and stage-specific expression of BmCHSA-2b in the silkworm, Bombyx mori
Source: Epigenetics Chromatin. 2018 Jun 14;11:32. doi: 10.1186/s13072-018-0202-4 (PMC6001065; doi:10.1186/s13072-018-0202-4)
Supplement: Supplementary file 10 — Additional file 10. Table. S1. The sequences of the primers in the study. The underlines represents the methylated sites. [file 13072_2018_202_MOESM10_ESM.pdf]

Primers	Sequence(5'-3')
**For qRT-PCR**	
BmCHSA -2a -F	AGGGAAACGGCGAGATGAG
BmCHSA -2a -R	CGATGAAGGTGACCAGGTAAGC
BmCHSA -2b -F	GAGACGGAGGAAGTCGTTTACA
BmCHSA -2b -R	TCCAAGGACCACGATGAAGG
BmDnmt1-F	CTCTGCGAGCTTTGTTGGACATG
BmDnmt1-R	CGCTGCCGCCAATATGATCAAC
BmDeaf1-F	CAGCATTGGAGAGAGTGAAGTGGAT
BmDeaf1-R	GTCTTGATTTCAGGGTTCCCCAGTA
SlCHSA -2b -F	CGTAAAAGTCACTATGAAAGGGACGATG
SlCHSA -2b -R	CAATAATGCAAAGTCCCATTGCTGTAA
SlDnmt1-F	AATCCGCTCCTAGACGAACGTGTA
SlDnmt1-R	GGAAATGGCTCTCTGGCATGGA
SlDeaf1-F	TACGCCAGCCGAACTTCATAA
SlDeaf1-R	ACTAGGAGTGTACCATTCCATTCCG
BmRP49-F	CAGGCGGTTCAAGGGTCAATAC
BmRP49-R	TACGGAATCCATTTGGGAGCAT
SlRP49-F	CGTGAGCTGGAGATCCTGATGAT
SlRP49-R	CTCTACAATGGTCTTGCGCTTCT
**For methylation detection (*BmCHSA-2b*)**	
BmCGI2-wai-F	GTTGTTTATTGTTAAGTATTTTTATAA
BmCGI2-wai-R	ATAACTTCTCACACACAAAAAC
BmCGI2-nei-F	TAAGTTTTAAAATATTGTAAGTTTAGTT
BmCGI2-nei-R	ATCAAATCAACATAAATTTAATTTATAT
BmCGI3-wai-F	TAATATAAATTAAATTTATGTTGATTTG
BmCGI3-wai-R	CCAAATATATCCATAACTCTCTATTATA
BmCGI3-nei-F	AAATTTATGTTGATTTGATAAATTT
BmCGI3-nei-R	ACCCAAAAAACAATTTTATATTT
BmCGI4-wai-F	GGAGGTTATTGTATAATTTTTAGTTG

BmCGI4-wai-R	TACAAACAATCACAACACTAACATC
BmCGI4-nei-F	TATTAGTATTTATGTAAAATATTTTTATT
BmCGI4-nei-R	TTTTTATAACAAACAAATATCCTATC
**For Promoter Reporter Construction(*BmCHSA-2b*)**	
CHSA-2b-CG3-core promoter -F	CTCGAGCTCGTGACCAGTGCCGCAGAACCAAGATACCGCTGCAC
CHSA-2b-mutCG3-core promoter -F	CTCGAGCTAGTGACCAGTGAAGCAGAACAAAGATACAGCTGCAC
CHSA-2b-core promoter -R	AGATCTAGCCGAGAATATCAATAAAATAGG
**For protein expression**	
Deaf1-GFP-F	CCGCGGATGGCCGAGAATAGAAGTTCGGACAACGTCGTC
Deaf1-GFP-R	GGATCCAGGTGCTGGCTTTCAACAATCAG
Dnmt1-GFP-F	GGTACCGATGCCCACTTCAACTATTACTTGC
Dnmt1-GFP-R	GGATCCCGCACTGTCGTGCTGAAGTACC
Deaf1-F	GGATCCATGGCCGAGAATAGAAGTTCGG
Deaf1-R	CTCGAGTTAGTGCTGGCTTTCAACAATCAGC
Dnmt1-GFP-F	GGATCCTGCCATGGTGCTCCGCTTTC
Dnmt1-GFP-R	CTCGAGATCTGTTCTTCCAACACTGATGGCA
**For RNAi**	
RNAi Deaf1-F	GAGGGCACGTCTGTGTTGTCTAC
RNAi Deaf1-R	TGTTCGCAGTCTCCGTCCAT
RNAi Deaf1-T7 F	TAATACGACTCACTATAGGGAGGGCACGTCTGTGTTGTCTAC
RNAi Deaf1-T7 R	TAATACGACTCACTATAGGTGTTCGCAGTCTCCGTCCAT
RNAi Dnmt1-F	ATCAAGCTGGAGTTGCAGAATG
RNAi Dnmt1-R	TTAAAGGCCACAAAGTTACGAA
RNAi Dnmt1-T7 F	TAATACGACTCACTATAGGATCAAGCTGGAGTTGCAGAATG
RNAi Dnmt1-T7 R	TAATACGACTCACTATAGGTTAAAGGCCACAAAGTTACGAA
dsGFPF	TACGGCGTGCAGTGCTTCAGCC
dsGFPR	GTGCTCAGGTAGTGGTTGTCGG

dsGFPF-T7	TAATACGACTCACTATAGGTACGGCGTGCAGTGCTTCAGCC
dsGFPR-T7	TAATACGACTCACTATAGGGTGCTCAGGTAGTGGTTGTCGG
**For EMSA**	
CG island 3-F	CTCGTGACCAGTGCCGCAGAACCAAGATACCGCTGCAC
CG island 3-R	GTGCAGCGGTATCTTGGTTCTGCGGCACTGGTCACGAG
CG island 3 mut-F	CTAGTGACCAGTGAAGCAGAACAAAGATACAGCTGCAC
CG island 3 mut-R	GTGCAGCTGTATCTTTGTTCTGCTTCACTGGTCACTAG
CG island 3 met-F	CTCGTGACCAGTGCCGCAGAACCAAGATACCGCTGCAC
CG island 3 met-R	GTGCAGCGGTATCTTGGTTCTGCGGCACTGGTCACGAG
**For ChIP**	
CGI3-ChIP-F	CGGCAATTAACGAGTGGACGATAT
CGI3-ChIP-R	CAGTAGTATCTTGGTTCTGCGACAC
